# Design, synthesis, and anticancer evaluation of a novel imine-linked covalent organic framework correlated to molecular docking and DFT insights

**DOI:** 10.1039/d6ra00306k

**Published:** 2026-04-10

**Authors:** Eman Abdelnasser, Esam S. Allehyani

**Affiliations:** a Chemistry Department, Faculty of Science, Kafrelsheikh University El-Geish Street, P.O. Box 33516 Kafrelsheikh Egypt eman_ahmed2014@sci.kfs.edu.eg; b Department of Chemistry, University College in Al-Jamoum, Umm Al-Qura University Makkah Saudi Arabia

## Abstract

A new imine-linked covalent organic framework (COFTDTSC) was synthesized from terephthaldehyde and thiosemicarbazide under solvothermal conditions. The obtained material exhibited excellent chemical and thermal stability up to 625 °C, along with a high surface area of 1097.76 m^2^ g^−1^ and an average pore size of ∼1.18 nm. Scanning electron microscopy (SEM) revealed an irregular, stone-like morphology distinct from the starting materials, confirming successful polymerization and framework formation. The anticancer potential of COFTDTSC was evaluated against three human cancer cell lines (HCT-116, HepG-2, and MCF-7), demonstrating notable cytotoxic activity with IC_50_ values ranging from 10.28 to 18.04 µM, in comparison with the standard drugs doxorubicin and sorafenib. Molecular docking supported these results, revealing that COFTDTSC had the strongest binding affinity (−9.1 kcal mol^−1^) and stable interactions within human topoisomerase IIα, supported by multiple hydrogen bonding and hydrophobic interactions within the active site. Density Functional Theory (DFT) calculations further examined the electronic structure and reactivity of the monomers and the COF. Polymerization into COFTDTSC reduced the energy gap and increased electronic softness, enhancing molecular reactivity. The COF exhibited delocalized charge density, a moderate dipole moment, and strong electron-acceptor properties, suggesting potential for biological interactions, such as DNA binding and enzyme inhibition. From a green chemistry perspective, the design of COFTDTSC integrates bioactive building blocks within a stable, porous organic framework, offering a promising platform for sustainable anticancer material development. These findings highlight COFTDTSC as a multifunctional candidate for future biomedical applications.

## Abstract 2

COFTDTSC: thermally stable covalent organic framework with potent anticancer activity.

## Graphical abstract

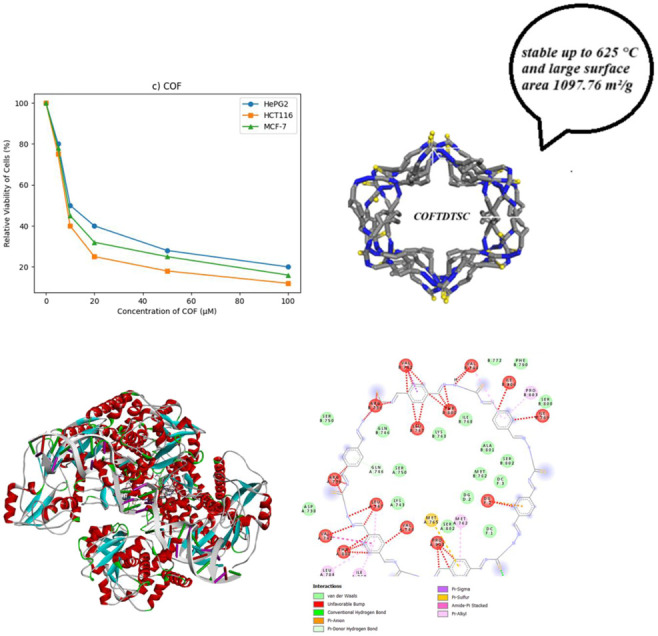

## 1. Introduction

Covalent organic frameworks (COFs) represent a rapidly developing class of crystalline, porous polymers. Built from lightweight elements (*e.g.*, C, H, N, O, S, B) connected by robust covalent bonds, they form extended two- or three-dimensional structures. Their appealing properties, such as adjustable porosity, large surface areas, structural integrity, and the capacity for functional group integration, make them excellent candidates for biomedical uses, including drug delivery, biosensing, and cancer treatment.^1–13^ Unlike traditional porous materials such as metal organic frameworks (MOFs) and zeolites, COFs offer the advantage of modular design, enabling precise tailoring of their pore environment and surface functionality at the molecular level. Recent studies have highlighted the potential of COFs as anticancer platforms due to their ability to encapsulate,^14,15^ stabilize, and release therapeutic agents in a controlled manner.^16–25^

In recent years, increasing attention has been directed toward the development of COF-based systems within the framework of green and sustainable chemistry. The principles of green chemistry emphasize the design of materials that minimize environmental impact, reduce toxicity, and enhance efficiency in chemical processes. COFs are particularly promising in this regard due to their metal-free composition, chemical stability, and potential for recyclability. Recent studies have highlighted the role of functional organic frameworks in sustainable catalysis, eco-friendly synthesis, and biologically active systems, demonstrating their relevance in advancing green chemical technologies.^26–28^

The incorporation of biologically active moieties into COF architectures has opened new opportunities for biomedical applications, particularly in anticancer therapy. Functional groups such as hydrazides, carbazides, and thiosemicarbazide introduce additional active sites capable of hydrogen bonding, metal coordination, and interactions with biomolecular targets. These features enhance the biological performance of COFs, enabling applications in drug delivery, enzyme inhibition, and DNA interaction. Moreover, recent reports have demonstrated that the integration of nitrogen- and sulfur-containing ligands notably improves the pharmacological activity and selectivity of organic frameworks, aligning with sustainable design strategies in medicinal chemistry. Furthermore, the inherent biocompatibility and biodegradability of organic building blocks can reduce systemic toxicity compared with inorganic nanocarriers. Functionalization with heteroatom-containing ligands, enhances COF bioactivity by introducing additional binding sites and facilitating interactions with biomolecules.^29^ One promising synthetic approach involves the condensation of terephthaldehyde with thiosemicarbazide, forming imine COFs with extended conjugation and electron-rich functionalities. These functional groups not only stabilize the COF framework but also provide sites for hydrogen bonding, metal chelation, and potential interactions with nucleic acids or proteins in cancer cells.^30^ The introduction of thiosemicarbazide units is particularly important, as these moieties are known to exhibit inherent anticancer properties, including DNA binding and inhibition of tumor cell proliferation.^31^ Incorporating them into COF scaffolds thus combines structural stability with therapeutic functionality, offering a synergistic strategy for designing next-generation anticancer nanomaterials. The present work aims to design and investigate a thiosemicarbazide-based covalent organic framework synthesized from terephthaldehyde and thiosemicarbazide (or analogous linkers), with a focus on its structural characteristics, functional group distribution, and potential anticancer applications. By integrating anticancer-active organic motifs into a porous COF backbone, this study seeks to provide a foundation for developing multifunctional therapeutic materials with enhanced efficacy and selectivity against cancer cells.

## 2. Materials and methods

### 2.1. Chemicals

Terephthaldehyde, benzaldehyde, thiosemicarbazide, acetic acid, 1,4-dioxane, and ethanol were purchased from Aldrich. Distillation was utilized to purify the organic solvent employed in this work, and it was then dried in a nitrogen atmosphere. Sigma (St. Louis, USA) provided the RPMI-1640 medium, MTT, and DMSO, and GIBCO (UK) provided the fetal bovine serum. The reference anticancer drugs used for comparison were sorafenib and doxorubicin.

### 2.2. Model compound synthesis

Benzaldehyde (0.203 mL, 2 mmol) was dissolved in 20 mL of 1,4-dioxane with stirring. Thiosemicarbazide (0.091 g, 1 mmol) was then introduced, and the mixture was heated under reflux for 48 hours. The reaction of thiosemicarbazide with two equivalents of benzaldehyde afforded the corresponding bis-thiosemicarbazone derivative. The resulting product was isolated by cooling the solution to room temperature, followed by filtration, thorough washing with methanol, and drying under reduced pressure. IR (KBr, cm^−1^): 3400–3200 (NH), 1100–1250 (C=S); ^1^H-NMR (400 MHz, DMSO-*d*_6_, *δ*, ppm): 8.5(H, s, CH=CN), 10.5(H, s, CH=CN), 7.00–7.60 (10H, m, Ar–H); 11.5(H, s, NH); ^13^C-NMR (400 MHz, DMSO-*d*_6_, *δ*, ppm): 140–150 (C–Ar), 155, and 158.25 (C=N),178 (C=S).

### 2.3. Synthesis of COFTDTSC

The COFTDTSC framework was synthesized under solvothermal conditions *via* a Schiff base reaction. Terephthaldehyde (1 mmol, 0.134 g) and thiosemicarbazide (1 mmol, 0.091 g) were dissolved in 30 mL of dioxane, with a few drops of acetic acid (9 M) added as a catalyst. The mixture was placed in a 10 mL Pyrex tube, subjected to three freeze–pump–thaw cycles for degassing, sealed under vacuum, and heated at 120 °C for 9 hours. A yellow precipitate formed upon cooling, which was collected by filtration and washed extensively with ethanol, methanol, diethyl ether, and acetone to yield the final COFTDTSC product (Scheme 2).

### 2.4. Instrumentation

Nuclear Magnetic Resonance (NMR) spectra (^1^H and ^13^C) were recorded on a Bruker DPX 400 MHz spectrometer, using tetramethyl silane (TMS) as an internal standard. Infrared (IR) spectra were obtained on a Thermo Fisher Scientific spectrometer using KBr pellets in the range of 400–4000 cm^−1^.

X-ray diffraction (XRD) patterns were collected on a PertPro diffractometer with Cu Kα radiation (*λ* = 1.5404 Å), scanning from 4° to 80° (2*θ*) at 25 °C. UV-visible absorption spectra were measured from 200 to 800 nm using a PerkinElmer Lambda 950 spectrophotometer.

Thermogravimetric analysis (TGA) was conducted with an SDT analyzer under a nitrogen atmosphere, heating samples from room temperature to 1000 °C at 10 °C min^−1^. Surface area and porosity were determined by nitrogen adsorption–desorption at 77 K using a JW-BK 132F analyzer. The surface morphology was examined with a JEOL JSM-IT100 scanning electron microscope (SEM), with samples prepared on gold-coated stubs after degassing at 120 °C for over 10 hours.

### 2.5. Anticancer activity

#### 2.5.1. Cell line

The human cancer cell lines used, hepatocellular carcinoma (HepG2), colorectal carcinoma (HCT-116), and breast adenocarcinoma (MCF-7) were supplied by the American Type Culture Collection (ATCC) *via* VACSERA, Cairo, Egypt.

#### 2.5.2. MTT assay

Cytotoxicity was evaluated using the MTT colorimetric assay, which measures the reduction of yellow MTT tetrazolium salt to purple formazan by mitochondrial dehydrogenases in viable cells. Cells were cultured in RPMI-1640 medium supplemented with 10% FBS and antibiotics at 37 °C in a 5% CO_2_ atmosphere. For the assay, 1.0 × 10^4^ cells per well were seeded in a 96-well plate and allowed to adhere for 48 hours. The cells were then treated with various concentrations of the test compounds for 24 hours. Subsequently, 20 µL of MTT solution (5 mg mL^−1^) was added to each well, and incubation continued for 4 hours. The formazan crystals formed were dissolved in 100 µL of DMSO, and absorbance was measured at 570 nm using a microplate reader (EXL 800, USA).

#### 2.5.3. Calculation of cell viability and inhibition percentage

Cell viability was calculated using the formula:Cell viability (%) = (absorbance of treated cells/absorbance of control cells) × 100

The inhibition percentage was derived by subtracting the viability percentage from 100%. All tests were performed in triplicate, and results are presented as mean ± standard deviation (SD). The IC_50_ values were calculated from dose–response curves using nonlinear regression.

### 2.6. Molecular docking studies

Molecular docking simulations were performed using the MOE software.^32^ The geometry of COFTDTSC was optimized, and a conformational search was conducted to an RMS gradient of 0.01 Å, with energy minimization carried out using AutoDock Vina. The target protein was the Cryo-EM structure of the Human topoisomerase II alpha DNA-binding/cleavage domain (PDB ID: 6ZY6).^33^ Ten independent docking runs were performed using default parameters, with conformations evaluated based on binding energy and interactions with key amino acids in the binding pocket.

### 2.7. Computational investigation

The theoretical investigation employed the DFT method with the WB97XD functional,^34^ and a 6-311(G) basis set, carried out *via* the Berny method using the Gaussian 09W software.^35^ Geometry optimization was performed without imposing symmetry constraints. To analyze vibrational modes, Vibrational Energy Distribution Analysis (VEDA) was utilized, which facilitated the determination of Potential Energy Distribution (PED) and enabled comprehensive assignments of vibrational modes.^36,37^

To accurately depict the electronic structure and reactivity of COFTDTSC, a fragment of the overall crystal structure was modeled rather than the complete periodic system. Full optimization of extended COF models necessitates DFT techniques utilizing plane-wave codes—such as VASP, CASTEP, and CP2K—which exceed the capabilities of Gaussian 09W. Consequently, a smaller oligomer fragment featuring multiple imine linkages (–C=S–NH–) was employed to represent COFTDTSC. This approach effectively simulated the local bonding conditions, conjugation pathways, and electronic distributions characteristic of the extended COF sheets.

The oligomeric representation preserved the essential electronic characteristics of the imine linkages and the aromatic framework, enabling feasible molecular DFT calculations. By focusing on a fragmented representation rather than an entire periodic structure, the authors could effectively model the electronic structure and reactivity of COFTDTSC. As previously mentioned, full periodic DFT optimization of extended COFs can only be achieved using plane-wave codes like VASP, CASTEP, and CP2K, which are not compatible with Gaussian 09W.

To develop a suitable fragment model for COFTDTSC, the authors created an oligomeric structure composed of imine-linked repeating units connected by (–C=S–NH–) moieties. This design retains the crucial electronic properties of imine linkages and an aromatic framework, ensuring all necessary characteristics for molecular DFT analysis. The DFT output provided electronic descriptors, including EHOMO (Energy of the Highest Occupied Molecular Orbital), ELUMO (Energy of the Lowest Unoccupied Molecular Orbital), Δ*E*_g_, electronegativity (*χ*), chemical hardness (*η*), softness (*σ*), electrophilicity index (*ω*), and maximum electron transfer (Δ*N*_max_). Additionally, molecular electrostatic potential and electron density maps were generated to illustrate local electron-rich and electron-poor areas relevant to bacterial interactions. VEDA analysis also contributed support for vibrational mode assignments and energy decomposition. Although the COFTDTSC model is based on an oligomeric unit, the findings offer insights into the local electronic properties of the imine-linked framework, acknowledging that these results do not represent a complete periodic DFT calculation.

## 3. Results and discussion

### 3.1. Chemistry

**Scheme 1:**
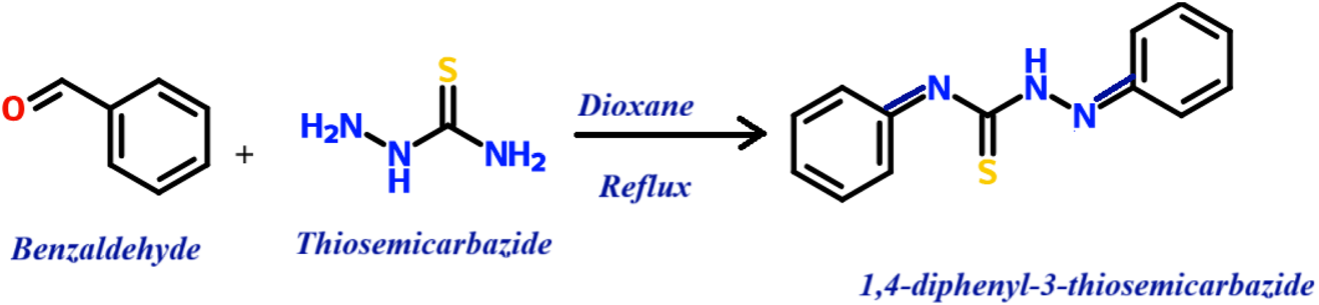
Model compound synthesis.

The COFTDTSC material was prepared under solvothermal condensation of terephthaldehyde with thiosemicarbazide, as illustrated in Scheme 1. In this process, the aldehyde and amine functional groups react *via* a Schiff base mechanism, generating stable imine (C=N) linkages that serve as the structural backbone of the framework. The formation of such linkages is characteristic of imine-linked covalent organic frameworks (COFs) and contributes significantly to their chemical robustness and structural regularity.

**Scheme 2:**
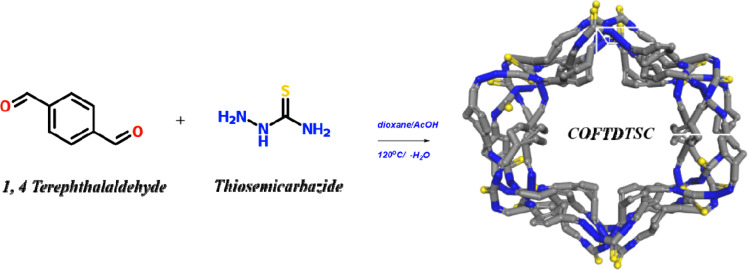
Synthesis of COFTDTSC through solvothermal conditions.

#### 3.1.1. FT-IR spectral analysis

**Fig. 1:**
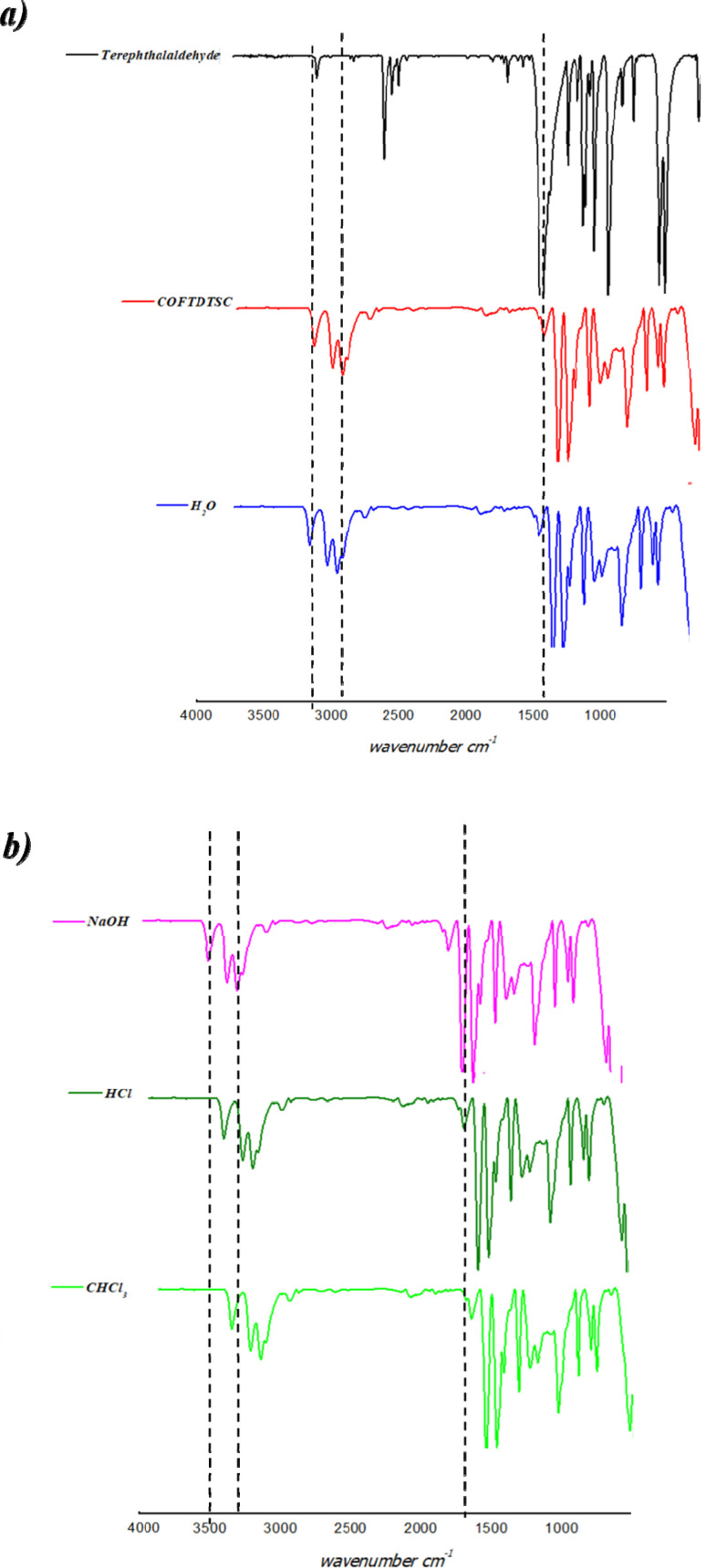
(a) Terephthaldehyde and COFTDTSC FT-IR spectral analysis; (b) COFTDTSC's chemical stability of COFTDTSC FT-IR spectrum under HCl, NaOH, CHCl_3_, and H_2_O.

The FT-IR spectrum confirmed the formation of COFTDTSC by the disappearance of the terephthaldehyde carbonyl stretch at ∼1685 cm^−1^. Broad bands in the 3280–3190 cm^−1^ region were assigned to NH groups, while aromatic C–H stretches appeared at 3090–2987 cm^−1^. Strong bands at 1592–1574 cm^−1^ corresponded to C=N stretching, and bands at 1255–1225 cm^−1^ were attributed to C=S vibrations. Peaks at 1102–1034 cm^−1^ were related to C–N stretches. The material was insoluble in common organic solvents and water. Stability tests in harsh conditions (6 M HCl, 6 M NaOH, CHCl_3_, H_2_O) for 14 days showed no structural degradation, as confirmed by unchanged FT-IR spectra, indicating high chemical stability (Fig. 1).

#### 3.1.2. UV-visible spectra analysis

**Fig. 2:**
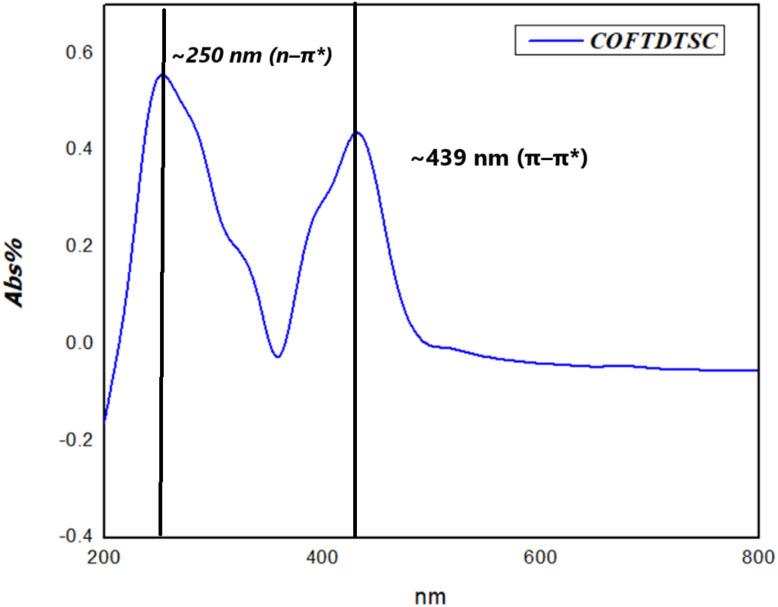
UV spectra of COFTDTSC.

UV-vis spectroscopy revealed electronic transitions in the framework (Fig. 2). Terephthaldehyde showed a π–π* transition peak at 298 nm. COFTDTSC exhibited two new bands at ∼250 nm (n–π*) and ∼439 nm (π–π*). The appearance of the red-shifted band beyond 300 nm indicates an extension of conjugation within the COFTDTSC structure, resulting from the formation of imine (C=N) linkages during the condensation process. This shift suggests enhanced electron delocalization across the framework, confirming successful construction of the extended conjugated COF network.

#### 3.1.3. ^1^H and ^13^C NMR and elemental analysis

**Fig. 3:**
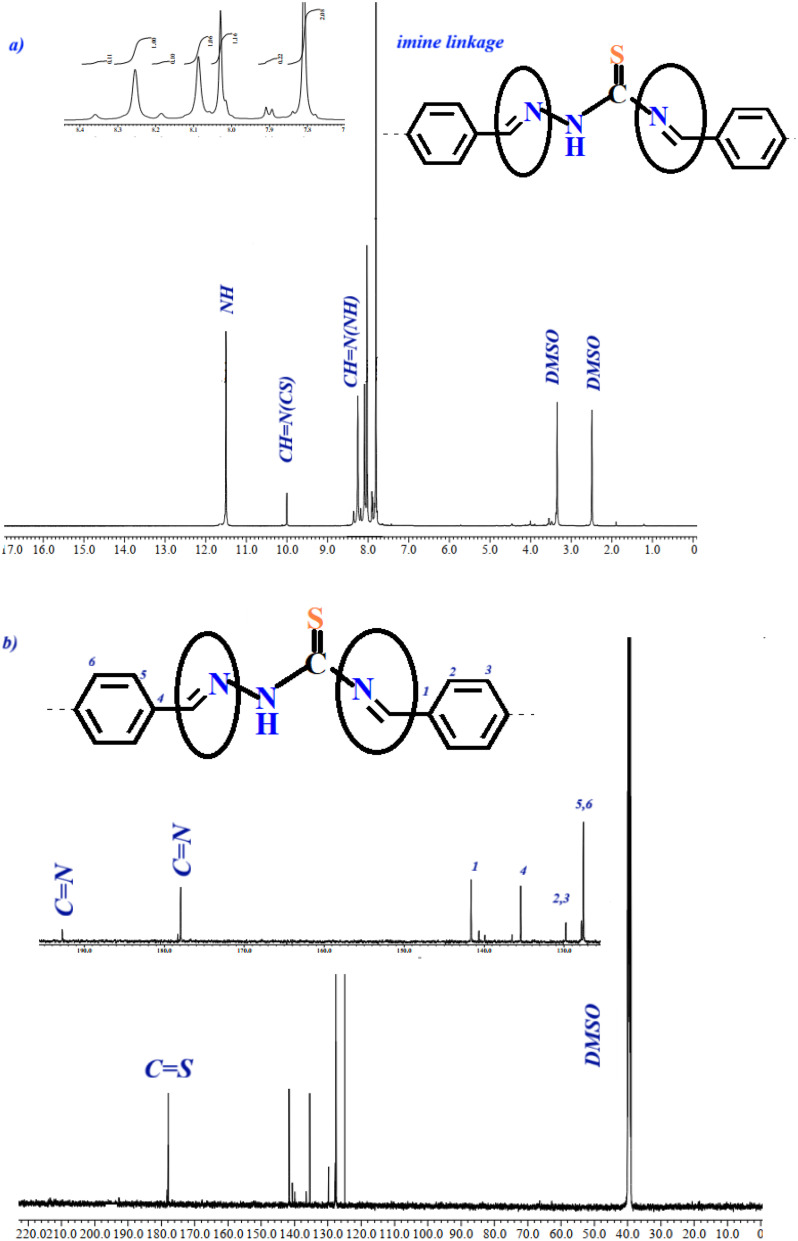
(a) ^1^H NMR and (b) ^13^CNMR of COFTDTSC.

The ^1^H NMR spectrum of COFTDTSC displayed a distinct singlet resonance at 9.90 ppm, corresponding to the proton of the imine (C=N) group, confirming the successful formation of imine linkages within the COF structure. Additionally, a broad signal observed at 6.74 ppm was assigned to the –NH proton, further supporting the incorporation of thiosemicarbazide units into the framework (Fig. 3a).

The ^13^C NMR spectrum (Fig. 3b) exhibited a characteristic signal at 158.25 ppm, attributed to the carbon of the imine (C=N) linkage. The disappearance of the carbonyl carbon signal of terephthaldehyde, previously appearing at around 191.10 ppm, further substantiates that the condensation proceeded *via* a Schiff-base mechanism, leading to successful framework formation.

Elemental analysis of COFTDTSC yielded values of C, 57.80%; H, 3.69%; and N, 19.42%, which are in close agreement with the theoretical values calculated for the proposed two-dimensional framework (C, 71.61%; H, 7.51%; N, 20.88%). These findings collectively confirm the successful synthesis of an imine-linked covalent organic framework with the expected chemical composition.

#### 3.1.4. Powder X-ray diffraction of COFTDTSC

**Fig. 4:**
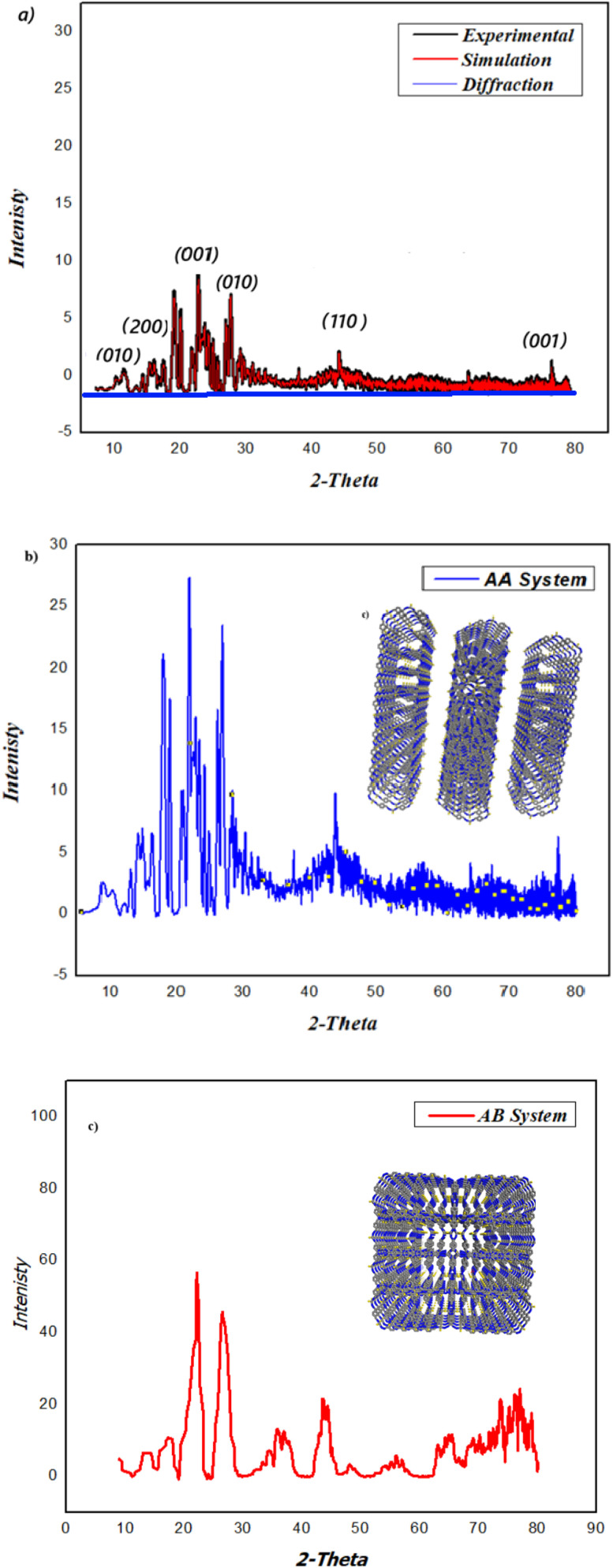
(a) Experimental and simulated PXRD patterns of COFTDTSC. The experimental pattern (black) shows good agreement with the simulated pattern (red), confirming the formation of a crystalline framework. The observed diffraction peaks are indexed to their corresponding Miller planes. The structural model is consistent with an AA (eclipsed) stacking arrangement, as evidenced by the close matching of peak positions and intensities; (b) simulated AA structure; (c) simulated AB structure.

PXRD analysis confirmed the crystalline structure of COFTDTS. Distinct diffraction peaks were observed at 2*θ* = 22.4° and 26.9°, along with a weak reflection at 2*θ* ≈ 6.34°, which corresponds to the (100) plane and indicates the porous architecture of the framework. A broad diffraction feature at 2*θ* ≈ 22.4° was assigned to π–π stacking interactions between adjacent COF layers (Fig. 4). The interlayer spacing, derived from Bragg's law, was calculated to be 3.8 Å, which is smaller than that reported for analogous 2D COFs,^38–40^ suggesting tighter packing of the stacked layers.

The PXRD pattern shows reasonable agreement between the experimental and simulated data. The main diffraction peaks were indexed to the (200), (010), (110), (300), (210), and (001) planes. The presence of a distinct (001) reflection at ∼27° indicates an ordered π–π stacking along the *c*-axis. The good agreement between experimental and simulated patterns suggests that the structure most likely adopts an AA stacking mode rather than AB stacking.

Pawley refinement of the experimental data (Fig. 4a) yielded satisfactory agreement factors (*R*_wp_ = 5.33%, *R*_p_ = 3.92%). The optimized structural model, based on geometrical energy minimization (Materials Studio v7.0), was indexed in the *P*222 space group with lattice parameters *a* = 31.339 Å, *b* = 15.371 Å, *c* = 14.227 Å, *α* = *β* = *γ* = 90°, closely matching the experimental structure. Simulated PXRD patterns for both the AA eclipsed and AB staggered stacking arrangements (Fig. 4b and c) reproduced the main features of the experimental diffraction profile, confirming the layered stacking mode of COFTDTSC.

#### 3.1.5. Thermal stability (TGA) for COFTDTSC

**Fig. 5:**
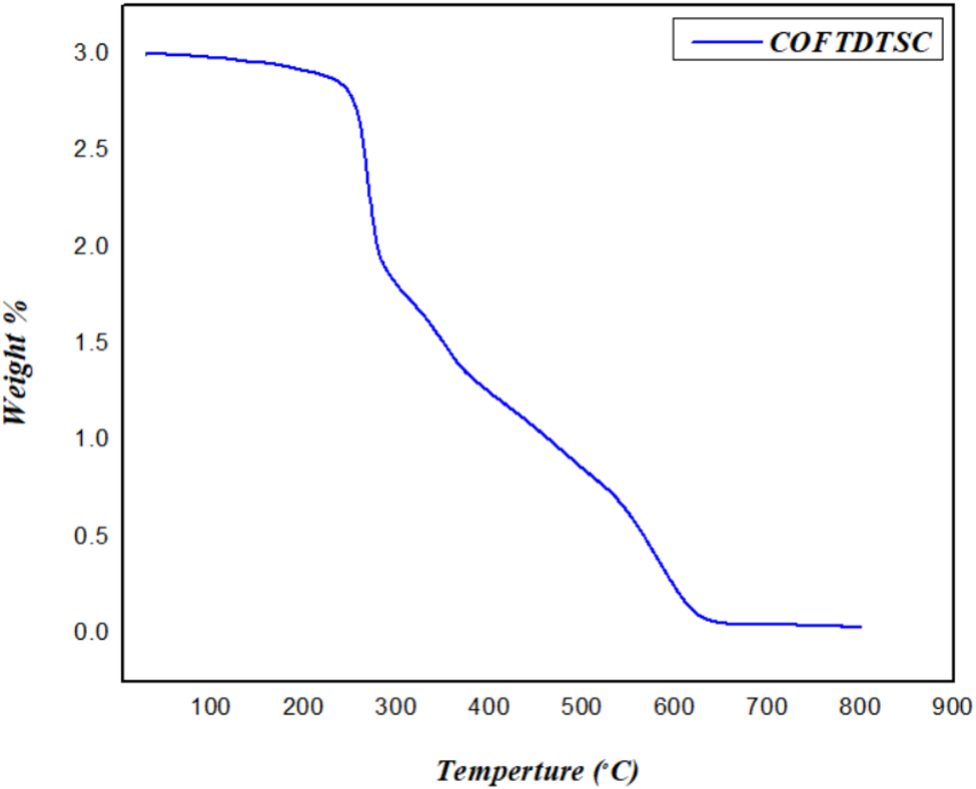
TGA curve of COFTDTSC.

TGA under nitrogen atmosphere demonstrated the framework's remarkable stability up to 625 °C (Fig. 5), suggesting strong covalent bonding within the framework, which was comparable to similar covalent organic frameworks previously reported in the literature, confirming the enhanced structural robustness of COFTDTSC. MCOFs, TpPa-1, ACOF-1, COF-JLU2.^41–43^

#### 3.1.6. Scanning electron microscope (SEM)

**Fig. 6:**
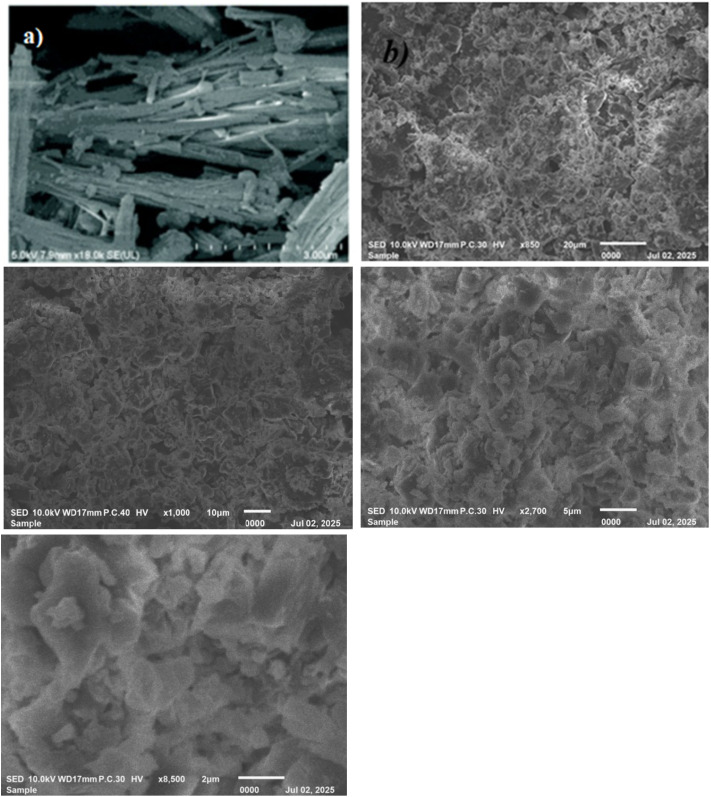
(a) SEM of terephthaldehyde; (b) SEM images of COFTDTSC at different magnifications showing the surface morphology and particle size distribution.

The surface morphology of the synthesized COFTDTSC was examined using scanning electron microscopy (SEM) at a magnification of 140k. As illustrated in Fig. 6b, the COFTDTSC sample exhibited an irregular, stone-like surface texture, markedly distinct from that of the terephthaldehyde precursor, which displayed elongated rod- and needle-shaped structures (Fig. 6a).^29^ The pronounced difference in surface features clearly indicates that the condensation and polymerization between terephthaldehyde and thiosemicarbazide were successfully achieved, resulting in the formation of the COFTDTSC framework.

#### 3.1.7. Adsorption isotherm

**Fig. 7:**
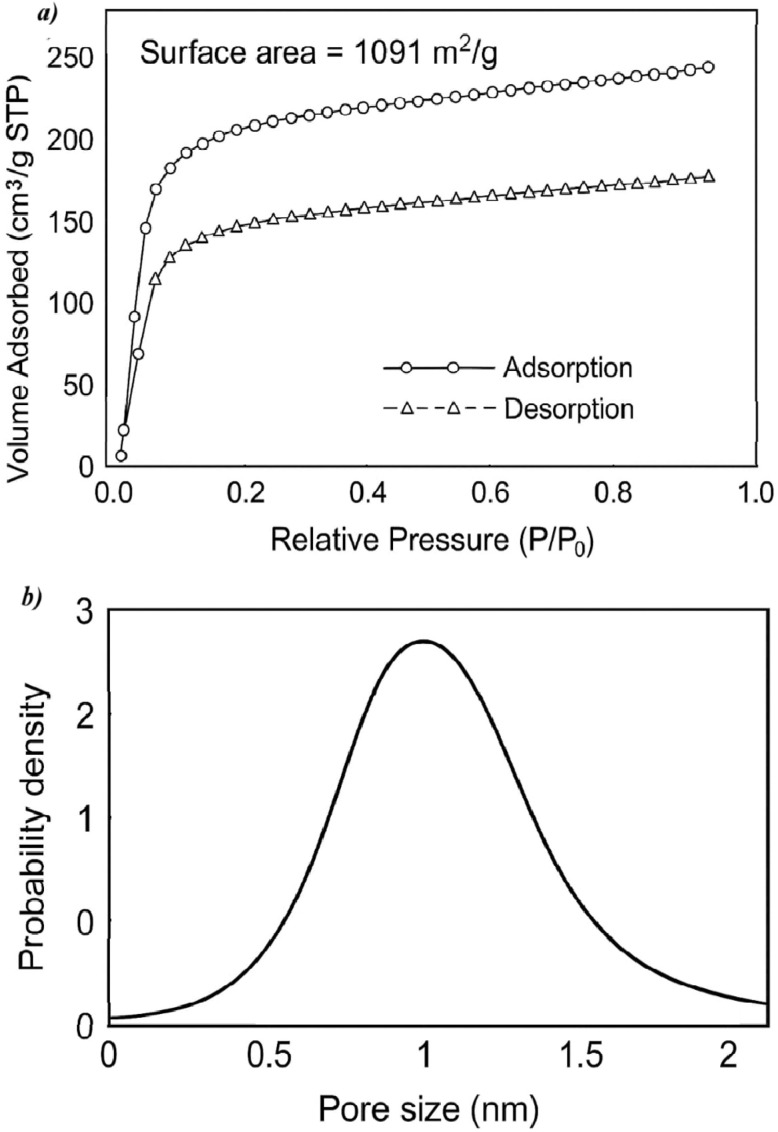
(a) N_2_ adsorption–desorption isotherm of COFTDTSC; (b) pore-size distribution profile.

Nitrogen adsorption–desorption analysis at 77 K was theoretically simulated to investigate the porous characteristics of the material. The resulting isotherm showed a Type IV isotherm (Fig. 7a), characteristic of microporous materials. The pore size distribution showed an average pore diameter of ∼1.18 nm (Fig. 7b). The BET surface area was calculated to be 1097.76 m^2^ g^−1^, with a total pore volume of 0.607 cm^3^ g^−1^, confirming a highly porous microstructure.

### 3.2. Biological evaluation

**Fig. 8:**
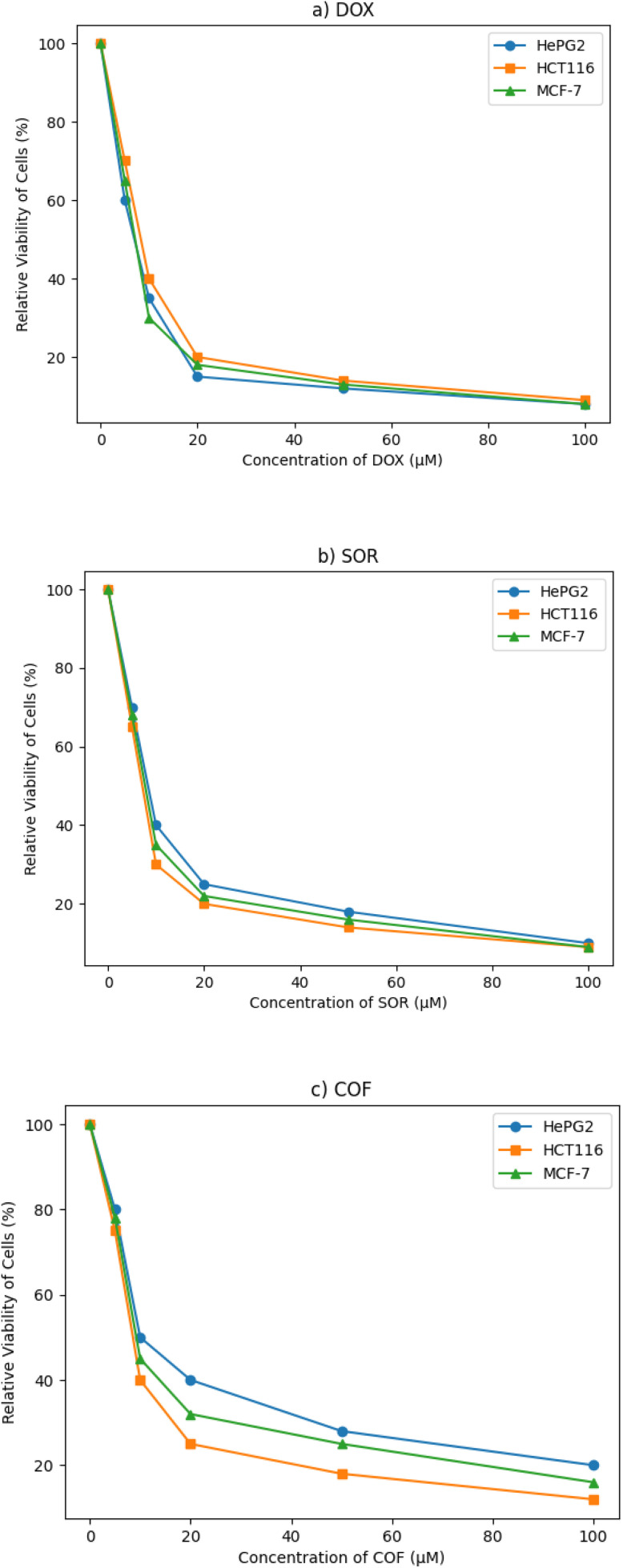
Dose–response curves for (a) DOX, (b) SOR, and (c) COFTDTSC.

The *in vitro* cytotoxicity of COFTDTSC was assessed against HePG-2, HCT-116, and MCF-7 cell lines, using DOX and SOR as references. DOX was the most potent, with IC_50_ values of 4.50 ± 0.2 µM (HePG-2), 5.23 ± 0.3 µM (HCT-116), and 4.17 ± 0.2 µM (MCF-7). SOR showed moderate activity (IC_50_ = 9.18 ± 0.6, 5.47 ± 0.3, and 7.26 ± 0.3 µM, respectively) (Table 1 and Fig. 8). COFTDTSC demonstrated considerable, though less potent, cytotoxic effects, with IC_50_ values of 18.04 ± 1.3 µM (HePG-2), 10.28 ± 0.8 µM (HCT-116), and 13.48 ± 1.1 µM (MCF-7), indicating a consistent antiproliferative profile across all cell lines. As shown in Table 1, COFTDTSC exhibits moderate cytotoxic activity compared to standard drugs such as DOX and SOR, while demonstrating comparable performance to several reported COF-based systems. This highlights its potential as a metal-free anticancer material COFTDTSC showed strong activity against three human cancer cell lines, HePG-2, HCT-116, and MCF-7, can be attributed to several mechanisms. First, a larger conjugated framework between (C=N), (C=S), (NH), and aromatic ring increases π-electron delocalization, improving compound stability and enhancing reactivity and potentially enabling π–π interactions with biological targets such as DNA bases. In addition, the presence of the –C=N– electron-rich and polar, making it a good site for nucleophilic or electrophilic attack. This imine linkage is crucial for coordination with metal ions and for hydrogen bonding with biomolecules. Moreover, the thioamide group (–C=S–NH–), the sulfur atom has a high polarizability and strong ability to coordinate with transition metals (Cu, Fe, Co, Pd, *etc.*), which may lead to ROS (reactive oxygen species) generation and enzyme inhibition, especially ribonucleotide reductase, which is a key anticancer mechanism. The C=S bond is more reactive than C=O due to its softer sulfur center promotes chelation and biological activity. Furthermore, intramolecular hydrogen bonding, the NH groups adjacent to the C=S or C=N centers, allows for stabilizing H-bonds, which may influence molecular geometry and electronic distribution, further enhancing reactivity.

**Table 1:** Comparison of cytotoxic activity (IC_50_, µM) of COFTDTSC with reported COF-based systems against HepG-2, HCT-116, and MCF-7 cancer cell lines

Comp.	*In vitro* cytotoxicity IC_50_ (µM)^a^			References
	HePG-2	HCT-116	MCF-7	
DOX	4.50 ± 0.2	5.23 ± 0.3	4.17 ± 0.2	This work
SOR	9.18 ± 0.6	5.47 ± 0.3	7.26 ± 0.3	This work
COF	18.04 ± 1.3	10.28 ± 0.8	13.48 ± 1.1	This work
TRIPTACISPLATIN	—	—	2.5	44
TRIPTA-COF	—	—	85	44
COF-FA@DOX	—	11.04 ± 0.85	—	45
COF@DOX	—	7.80 ± 1.92	—	45
Purp@COP	0.60	—	—	46

a IC_50_ (µM) scale: 1–10 (very strong); 11–20 (strong); 21–50 (moderate); 51–100 (weak); >100 (non-cytotoxic). DOX: doxorubicin; SOR: sorafenib.

### 3.3. Molecular docking studies

**Fig. 9:**
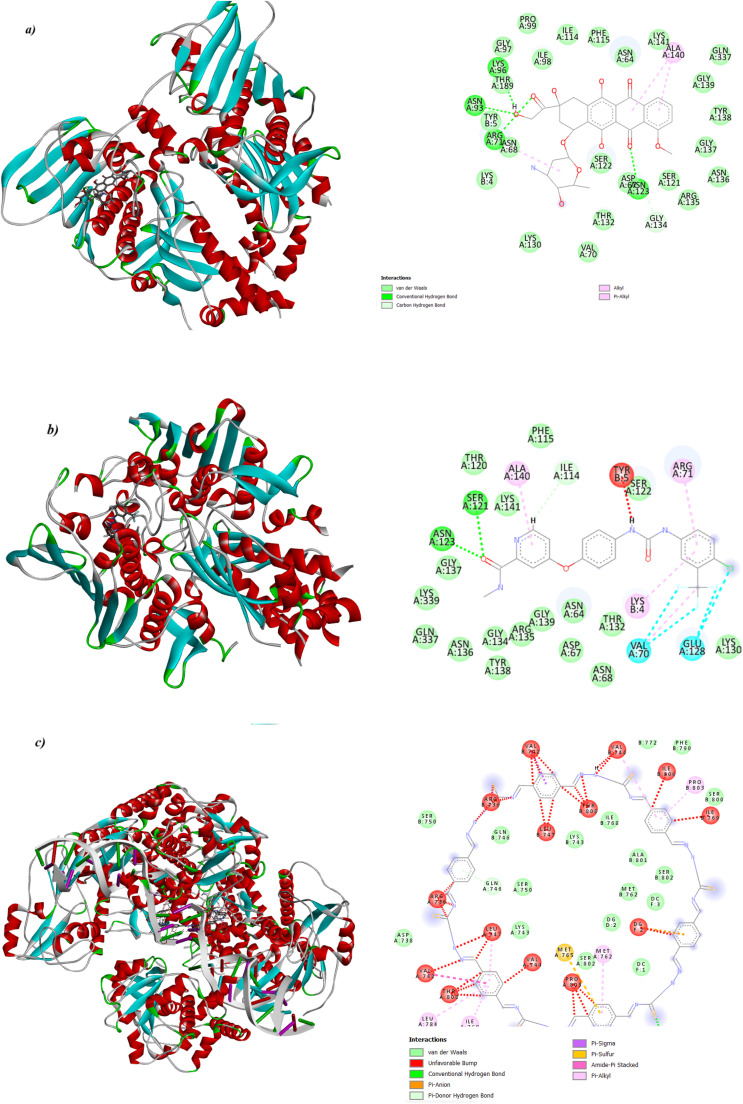
Binding modes of (a) DOX, (b) SOR, and (c) COFTDTSC with the human topoisomerase IIα (PDB ID: 6ZY6).

Docking simulations were conducted to explore potential binding modes and inhibitory effects. COFTDTSC exhibited the most favorable binding affinity (−9.1 kcal mol^−1^) compared to SOR (−8.3 kcal mol^−1^) and DOX (−7.5 kcal mol^−1^) (Fig. 9a–c), which may contribute to its observed cytotoxic activity. The interaction distances were shorter for COFTDTSC (2.5–3.0 Å) compared to SOR (3.1–3.6 Å), suggesting stronger hydrogen bonding and hydrophobic contacts. DOX interacted with residues like Ala, Asn, Ser, Arg, Asp, and Lys. SOR engaged a broader network including Ala, Ile, Asn, Gly, Ser, Glu, Val, Lys, Arg, and Phe. COFTDTSC formed an extensive and compact interaction pattern with residues Val, Leu, Tyr, Ser, Thr, Phe, Arg, Pro, and Ile. This binding profile suggests that COFTDTSC may interact effectively with the active site, suggesting its potential as a candidate for further investigation as a topoisomerase IIα inhibitor.

DOX formed multiple hydrogen bonds and electrostatic interactions with amino acid residues such as Ala, Asn, Ser, Arg, Asp, and Lys, contributing to moderate binding stability. SOR showed a broader interaction network involving Ala, Ile, Asn, Gly, Ser, Glu, Val, Lys, Arg, and Phe residues, reflecting its favorable accommodation within the binding pocket and enhanced stability through hydrophobic and π–π interactions. COFTDTSC displayed the most extensive and compact binding pattern, forming several hydrogen bonds and π-type interactions with residues including Val, Leu, Tyr, Ser, Thr, Phe, Arg, Pro, and Ile. These interactions, coupled with its shorter bond distances and higher binding energy, suggest that COFTDTSC has a strong molecular affinity and a well-fitted conformation within the topoisomerase IIα active site. Such binding characteristics highlight its potential as a promising lead compound for further optimization as a topoisomerase IIα inhibitor (Table 2).

**Table 2:** Molecular docking parameters of the tested compounds with human topoisomerase IIα (PDB ID: 6ZY6)

	Energy affinity (kcal mol^−1^)	Distance (Å)	Amino acids
DOX	−7.5	2.6–3.2	ALA, ASN, SER, ARG, ASP, LYS
SOR	−8.3	3.1–3.6	ALA, ILE, ASN, GLY, SER, GLU, VAL, LYS, ARG, LYS, PHE
COFTDTSC	−9.1	2.5–3.0	VAL, LEU, TYR, SER, THR, PHE, ARG, PRO, ILE

### 3.4. COFTDTSC computational studies

#### 3.4.1. Optimization of COFTDTSC

The electronic structures of terephthaldehyde, thiosemicarbazide, and the resulting COFTDTSC were analyzed to understand their frontier molecular orbitals and global reactivity parameters. Terephthaldehyde exhibits a relatively wide HOMO–LUMO energy gap (4.80 eV), indicating a chemically stable and electronically hard molecule. The high chemical hardness (*η* = 2.40 eV) and low softness (*σ* = 0.416 eV^−1^) suggest limited charge redistribution, consistent with the rigid and conjugated aromatic backbone of the aldehyde precursor. Its electrophilicity index (*ω* = 17.7 eV) reflects its strong tendency to accept electrons, driven by the electron-withdrawing aldehyde groups.

Thiosemicarbazide shows a significantly different behavior, characterized by a narrower gap and lower electronic hardness, which indicates higher reactivity and greater susceptibility to electronic perturbation. The presence of electron-rich nitrogen and sulfur atoms increases the electron-donating ability of the molecule, making it chemically softer and more nucleophilic. This enhanced softness supports its role as the nucleophilic linker during the formation of the COF framework.

COFTDTSC structure displays a band gap of ≈1.769 eV as estimated from periodic calculations, whereas the molecular DFT calculation on the oligomeric model yielded a smaller HOMO–LUMO gap of 1.769 eV due to finite size effects and enhanced electron delocalization, confirming the improved electronic delocalization resulting from extended π-conjugation throughout the 2D framework. Compared to the isolated monomers, the COF exhibits a considerable decrease in chemical hardness (*η* = 2.236 eV) and a substantial increase in softness (*σ* = 0.447 eV^−1^), demonstrating enhanced charge mobility within the periodic lattice. The electrophilicity index (*ω* = 5.498 eV) is notably lower than that of terephthaldehyde, indicating that the COF becomes less electron-accepting and more electronically balanced after polymerization. This shift reflects stabilization of the electronic density across the COF sheets.

The Mulliken charge distribution also supports this interpretation. In the monomers, the most negative charges are localized on heteroatoms (O and N), whereas in the COF, they become more evenly distributed, demonstrating the formation of an extended conjugated network with reduced localized electronic density. The dipole moment of COFTDTSC (1.48 D) lies between those of the two precursors, confirming the partial cancellation of dipole vectors upon framework formation.

Overall, the computational descriptors clearly show that the transformation from isolated reactive monomers to the COF structure results in a more stable, delocalized, and electronically uniform material. The narrower band gap and higher softness of COFTDTSC confirm its enhanced electronic communication and potential functional properties such as charge transport, adsorption, or photocatalytic behavior.

1 Δ*E* = *E*_LUMO_ − *E*_HOMO_

2
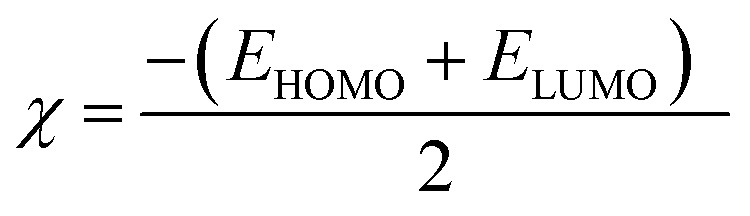
3
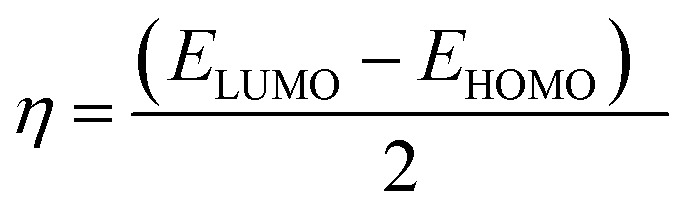

4 *σ* = 1/*η*

5 Pi = −*χ*

6 *S* = 1/2*η*

7 *ω* = Pi^2^/2

8 Δ*N*_max_ = −Pi/*η*

COF was used to test anticancer activity because the results showed that thiosemicarbazide exhibited significantly higher electronic flexibility, attributed to electron-rich nitrogen and sulfur atoms. These heteroatoms enhance the molecule's ability to donate or accept electrons, which is essential for biological mechanisms such as chelation of metal ions (*e.g.*, Cu^2+^, Fe^2+^) involved in cancer cell metabolism, ROS generation, or interaction with nucleophilic sites on DNA. The reduced hardness and increased softness of thiosemicarbazide suggest stronger biological reactivity, making it a suitable building block for anticancer agents.

**Fig. 10:**
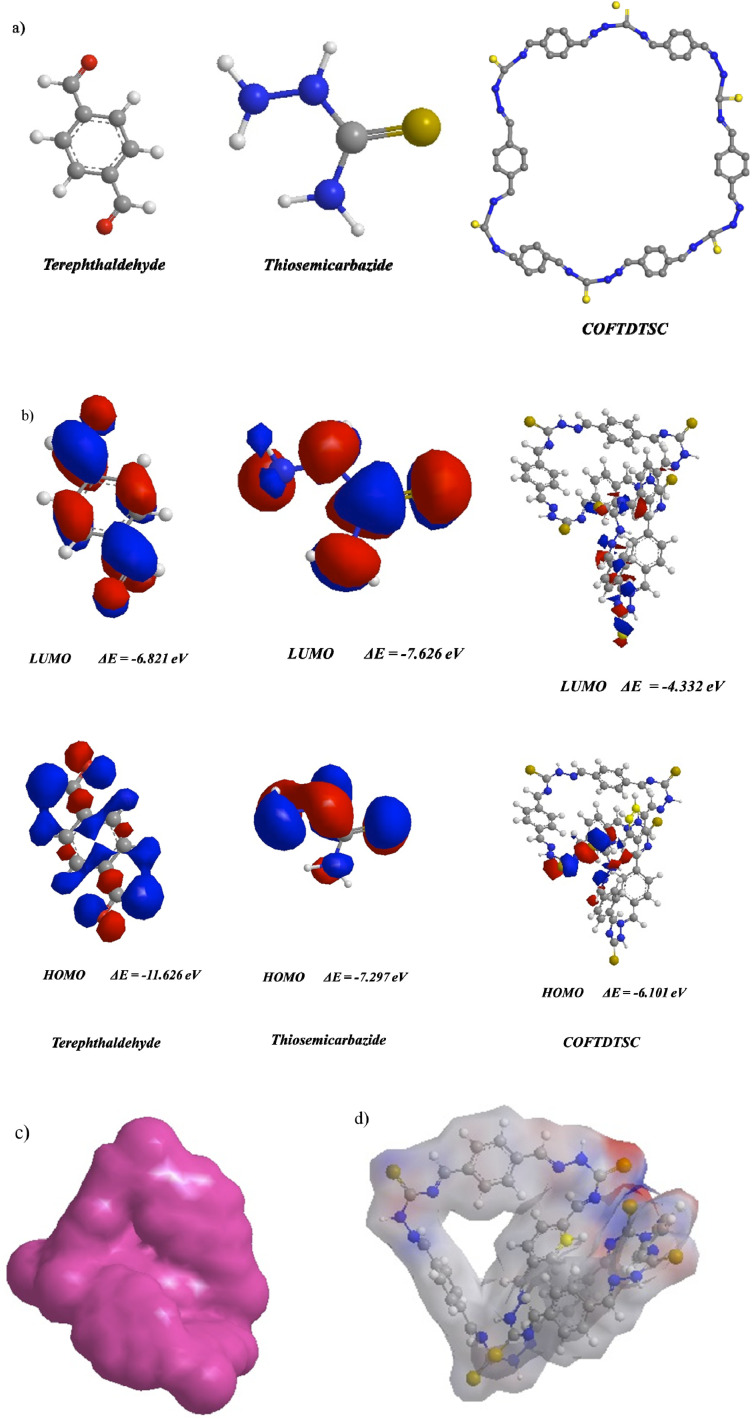
(a) Optimized molecular structure of terephthaldehyde, thiosemicarbazide, and COFTDTSC; (b) HOMO–LUMO of optimized structure; (c and d) ESP, MEP of COFTDTSC.

Upon polymerization, COFTDTSC framework exhibits a substantially reduced energy gap (1.769 eV) (Fig. 10 and Table 3) compared to terephthaldehyde, indicating improved electronic delocalization and increased chemical responsiveness. The enhanced softness (*σ* = 0.447 eV^−1^) and reduced chemical hardness (*η* = 2.236 eV) imply greater ease in electron transfer processes, an essential feature for anticancer materials capable of redox interactions or ROS-mediated cytotoxicity. The moderate electrophilicity index (*ω* = 5.498 eV) shows that COFTDTSC maintains a balanced ability to accept electron density from biomolecular targets such as DNA bases or protein thiol groups.

Ground-state energies of COFTDTSC, terephthaldehyde, and thiosemicarbazide utilizing DFT/B3LYP/6-311(G) and their physical parameters

**Table:** 

DFT/WB97XD/6-311(G)			
Physical descriptors	Terephthaldehyde	Thiosemicarbazide	COFTDTSC
*E*_T_ (au)	−458.679	−111.7924	−2505.32377
*E*_HOMO_ (eV)	−11.626	−7.292	−6.101
*E*_LOMO_ (eV)	−6.821	−7.626	−4.332
*E*_g_ (eV)	4.8050	14.923	1.7690
*µ* (D)	1.424	1.48	−5.4227
*χ* (eV)	9.2235	−0.1645	5.2165
*η* (eV)	2.4025	7.4615	0.8845
*σ* (eV)	0.4162	0.1340	1.1306
Pi (eV)	−9.2235	0.1645	−5.2165
*S* (eV)	0.2081	0.0670	0.5653
*ω* (eV)	17.7051	0.00181	15.3826
Δ*N*_max_	3.8391	0.0220	5.8977

**Table:** 

Net charge	O_16_	−0.345	N_1_	−0.587	N_57_	−0.298
	O_9_	−0.345	N_2_	−0.587	N_60_	−0.308
	C_7_	0.159	H_3_	0.312	S_1_	−0.245
	C_8_	0.159	H_4_	0.274	H_3_	0.312
	H_15_	0.146	H_5_	0.312	H_4_	0.274
	H_16_	0.146	H_6_	0.274	H_96_	0.197
					C_29_	0.024
					C_54_	−0.033
					C_59_	0.047

Furthermore, the charge distribution within COFTDTSC becomes more uniform compared to its monomers. This charge delocalization may facilitate π–π stacking interactions with nucleic acid bases, enhancing the COF's potential to intercalate into DNA a mechanism commonly associated with anticancer cytotoxicity. The moderate dipole moment (1.48 D) may also promote favorable interactions with biological membranes, improving cellular uptake.

Collectively, these computational findings indicate that COFTDTSC has a more reactive, electronically adaptable structure than its starting materials. The reduced band gap, increased softness, and balanced electrophilic behavior all support its potential biological activity, particularly in electron-transfer mechanisms, DNA binding, or oxidative stress induction, key pathways through which many anticancer agents exert their therapeutic effects.

## 4. Conclusion

The present study investigated the *in vitro* cytotoxic activity of COFTDTSC against three human cancer cell lines, HePG-2, HCT-116, and MCF-7, in comparison with the standard anticancer agents doxorubicin (DOX) and sorafenib (SOR). The results revealed that COFTDTSC exhibited moderate cytotoxic activity, with IC_50_ values higher than those of the reference drugs. Although its potency was lower, COFTDTSC demonstrated a consistent antiproliferative profile across all tested cell lines, indicating its potential as a lead structure for further chemical modification and optimization. Molecular docking studies supported these findings, showing that COFTDTSC exhibited favorable binding affinity (−9.1 kcal mol^−1^) and interaction patterns with key amino acid residues such as VAL, LEU, TYR, SER, THR, PHE, ARG, PRO, and ILE. These interactions may contribute to its observed biological activity compared to DOX (−7.5 kcal mol^−1^) and SOR (−8.3 kcal mol^−1^). Future studies should focus on structure–activity relationship (SAR) analysis, mechanistic investigations, and *in vivo* evaluation to better understand its therapeutic potential and enhance its anticancer efficacy. Computational analyses reveal that the transformation of terephthaldehyde and thiosemicarbazide into COFTDTSC results in significant enhancement of electronic softness, reduced HOMO–LUMO gap, and improved charge delocalization. These features collectively may support biological interactions, including DNA binding, enzyme inhibition, metal chelation, and ROS-mediated cytotoxicity. The electronic configuration and global reactivity descriptors of COFTDTSC indicate that it is a promising anticancer candidate with tunable electronic properties suitable for biomedical applications.

## Author contributions

Eman Abdelnasser – suggested idea, investigation, conceptualization, visualization, methodology, simulation, molecular docking, computational studies, writing – original draft, writing – review & editing. Esam S. Allehyani – resources, funding, validation, review & editing.

## Conflicts of interest

The authors declare that they have no known competing financial interests or personal relationships that could have appeared to influence the work reported in this paper.

## Data Availability

The data supporting this study are provided within the article.
